# APA-style human milk fat analogue from silkworm pupae oil: Enzymatic production and improving storage stability using alkyl caffeates

**DOI:** 10.1038/srep17909

**Published:** 2015-12-08

**Authors:** Xi Liu, Xudong Wang, Na Pang, Weijie Zhu, Xingyu Zhao, Fangqin Wang, Fuan Wu, Jun Wang

**Affiliations:** 1 School of Biotechnology, Jiangsu University of Science and Technology, Zhenjiang 212018, P R China; 2 Sericultural Research Institute, Chinese Academy of Agricultural Sciences, Zhenjiang 212018, P R China

## Abstract

Silkworm pupae oil derived from reeling waste is a rich source of *α*-linolenic acid (ALA), which has multipal applications. ALAs were added in *sn*-1, 3 positions in a triacylglycerol (TAG) to produce an APA-human milk fat analogues (APA-HMFAs, A: *α*-linolenic acid, P: palmitic acid). The optimum condition is that tripalmitin to free fatty acids of 1:12 (mole ratio) at 65 °C for 48 h using lipase Lipozyme RM IM. Results show that, the major TAG species that comprised APA-HMFAs were rich in ALA and palmitic acid, which contained 64.52% total unsaturated fatty acids (UFAs) and 97.05% PA at the *sn*-2 position. The melting point of APA was −27.5 °C which is much lower than tripalmitin (40.5 °C) indicating more plastic character. In addition, the practical application of alkyl caffeates as liposoluble antioxidants in APA was developed. Alkyl caffeate showed a superior IC_50_ (1.25–1.66 *μ*g/mL) compared to butyl hydroxy anisd (1.67 *μ*g/mL) and L-ascorbic acid-6-palmitate (L-AP) (1.87 *μ*g/mL) in DPPH analysis. The addition of ethyl caffeate to oil achieved a higher UFAs content (73.58%) at high temperatures. Overall, APA was obtained from silkworm pupae oil successfully, and the addition of caffeates extended storage ranges for APA-HMFAs.

Fats and oils are one of the most energy-rich food materials, which have the highest caloric values compared to other nutritional components^1^. Current processes for the production of structured triacylglycerols (TAGs) from vegetable and animal oil focus on enzymatic transesterification to create the novel fat replacements. TAGs and human milk fat substitutes have been synthesized by enzymatic catalysis in many studies^2^,^3^. Compared with the chemical methods, enzymatic approaches for lipid modification are more attractive due to the production of desirable acyl moieties or esters via specific enzymatic catalysis. Enzymatic processes are environmentally friendly and can be applied under mild conditions, ensuring greater product safety^2^. Currently, organic solvents with low water content are usually employed to improve enzyme performance^4^, which is important to protect or control acyl group migration to ensure a desired product synthesis^5^.

Human milk fat (HMF) is one of the major components of breast milk for newborn, term, and preterm infants. Thus, it supplies the highest fraction of an infant’s required dietary energy and nutrients^6^. The structure of HMF must be simulated to manufacture human milk fat analogues (HMFAs) for better digestion. The steric configuration of fatty acid is determined by chain length and unsaturated degree^7^, and polyunsaturated fatty acids have higher steric hindrance. It was not clear if the polyunsaturated fatty acid could be served as feedstock in the lipase-catalyed HMF production. Human milk is characterized by the dominance of TAGs (>98% of HMF), which contain palmitic acid (C16:0, 20–40% of total FA) in the *sn*-2 position (70% of all palmitic acid) and unsaturated fatty acids (UFAs) on *sn*-1 and *sn*-3 positions. Therefore, research on the synthesis of desirable structured TAGs focuses on the creation of TAGs rich in specific fatty acids in *sn*-2 position. Qin *et al.* investigated the incorporation of different fatty acids (C8:0-C18:2) into PPP-enriched TAGs to produce HMFA through lipase-catalyzed reactions^2^, and they also reported the degree of incorporation of different FAs into PPP-enriched TAGs through acidolysis catalyzed by lipase. Essential fatty acids, such as *α*-linolenic acid (ALA, C18:3, *ω*-3), from agricultural sources, may be used as the substrate to formulate APA-style HMFA for infant formula. A possible method would be to blend this product and 1, 3-dioleoyl-2-palmitoylglycerol (OPO) enriched fats and minor lipids based on the chemical composition of HMF.

Silkworm (*Bombyx mori* L.) pupae oil was obtained from desilked pupae, and identified as a good source of ALA. ALA is a principal omega-3 long-chain polyunsaturated fatty acid (*n*-3, LCPUFA) and an essential fatty acid in the human diet. Cardiovascular, hypertensive, inflammatory and autoimmune disorders can be effectively prevented by the administration of ALA^8^. ALA is regarded as the parent compound of the *n*-3 polyunsaturated fatty acid (PUFA) series, served as the precursor for all *n*-3 LCPUFA found in mammals^9^. In addition, ALA is a key precursor in converting to EPA and DHA which will contribute to the intellectual development of infants in the body^10^. In present, ALA was derived mainly from terrestrial plant sources such as flaxseed and perilla seed oils.

China is the largest producer of desilked silkworm pupae in the world. This kind of desilked pupae are mostly used as agricultural fertilizer and animal feed. Unfortunately, only small quantities of available pupae are used completely and large quantities are discarded as industrial waste^11^. ALA derived from silkworm pupae is simple in structure, highly efficient and reliable for the production of functional foods. Obtaining an ALA concentrate and transforming it into edible structured TAGs would be useful provided the conversion process could be performed at a reasonable price. To get TAGs with high palmitic acid content at *sn*-2 position, tripalmitin can be used as a carbon skeleton^12^. However, the application of this silkworm-derived products, has been hindered by oil oxidation, which is a critical problem in food industry that affects food quality. Thus, development of novel antioxidants is essential to prolong the oil storage time.

Synthetic liposoluble antioxidants, such as dibutyl hydroxy toluene (BHT) and butylated hydroxyanisole (BHA), are widely used in the edible oil industry due to their antioxidant properties. However, BHT and BHA have deleterious effects on human liver activity, spleen and lungs, as well as their ability to induce carcinomas^13^, have prompted researchers to study the screening healthier and greener antioxidants from natural products. Natural products have great promise in food industry applications as replacements for synthetic antioxidants in oily food^14^,^15^. Alkyl caffeates, which are synthesized from caffeic acid and various alcohols, have stronger lipid solubility in non-polar solvents than hydroxycinnamic acid. These compounds are more likely to be applied within oily foods as antioxidants due to antioxidative activity^16^. In addition, the potential bioactivity of alkyl caffeates have been investigated by several studies, indicating inhibition of lipopolysaccharide-induced nitric oxide production^17^, antinociceptive activity^18^, antitumor activities^19^, anticancer activity^20^ and the oxidation of edible oils^21^. The purpose of our work was to synthesize ALA-enriched structured TAGs using *sn*-1, 3-specific Lipozyme RM IM lipase through enzymatic interesterification of ALA from desilked silkworm pupae oil and glycerol PPP in an *n*-hexane system (see Fig. 1). Effects of different reaction conditions (include lipases, reaction time, temperature, and substrate molar ratio) on interesterification were investigated. To optimize reaction conditions, response surface methodology of the enzymatic reactions was investigated. The practical application of alkyl caffeates as liposoluble antioxidants in structured TAGs from desilked silkworm pupae oil was developed. Ten alkyl caffeates with varied chain lengths was explored to study the antioxidant activities on structured TAGs, prepared from desilked silkworm pupae oil. The properties of these compounds with respect to their hydroxyl radical-scavenging ability and effect on unsaturated fatty acids are also investigated.

## Results and Discussion

### Fatty acid profiles from desilked silkworm pupae oil

Based on GC analysis, all major components in the samples were separated from each other and identified. Desilked silkworm pupae oil was characterized as the initial material. Table 1 shows the FA compositions of the saponified silkworm pupae oil that was used to estimate the average molecular weight of the desilked silkworm pupae oil FFA (MW = 275.95 g/mol). The major FAs contents in silkworm pupae oil were ALA (34.73 ± 1.74%), oleic acid (31.16 ± 1.56%), palmitic acid (19.50 ± 0.97%), linoleic acid (7.55 ± 0.38%) and stearic acid (7.05 ± 0.35%). The saturated fatty acid content was 26.55%. Compared with other materials like olive oil and fish oil, silkworm pupae oil has more ALA which can serve as the DHA and EPA precursor in human body. In addition, the cost of materials containing DHA and EPA are much higher than silkworm oil containing ALA. The aim of this research was to produce structured TAGs with immobilized lipases by modifying the palmitin TAG, increase the UFAs, especially ALA esterified at the *sn*-1, 3 positions, and to physically and chemically characterize the resulting structured TAGs.

However, saturated fatty acids were not beneficial to the reaction. A rapid, simple and inexpensive procedure recently developed for urea fractionation was applied to isolate polyunsaturated FFA from the FFA extract derived from desilked silkworm pupae oil. This process removed saturated and monounsaturated FFA^22^,^23^. Table 1 shows the comparison of FFA content before and after the urea inclusion method from crude silkworm pupae oil, after the urea-based fractionation. These data were used to estimate the average molecular weight of the FFA (MW = 279.04  g/mol) after the process. The content of palmitic acid, stearic acid and oleic acid were decreased to 0.77%, 0% and 13.96%, respectively. The linoleic acid and ALA were increased to 12.10% and 73.16%. The total content UFAs reached to 99.22% after the urea-based fractionation, which was a favorable acyl donor for our further reactions.

Desilked silkworm pupae oil is a good source of ALA, but selectively enriching ALA is a technical challenge due to the presence of multiple analogues such as linoleic acid and oleic acid. A two-stage combinative inclusion process by *β*-cyclodextrin (*β*-CD) and urea was compared in Table 1, reported by Wang *et al.*^11^. Compared with a two-stage combinative inclusion process, a higher ALA content (73.16%) from crude oil of silkworm pupae was obtained by the urea inclusion method. Urea molecules readily form solid-phase complexes with saturated FFA, however, the characteristics in the hydrocarbon chains, such as double bonds, branching, or bulky constituents, greatly reduce the propensity for urea complex (UC) formation. PUFAs (e.g. C18:3) have been isolated through removal of saturated (e.g. C16:0) and monounsaturated FFA (e.g. C18:1) by UC formation.

Compared with other oils and fats, such as soybean oil (8.77% of ALA), the silkworm pupae is a superior source for ALA production. The FA content of these animal oils and fats is: lauric acid (0.10%), myristic acid (1.00%), PA (23.90%), stearic acid (15.40%), OA (41.60%), linoleic acid (13.20%) and ALA (0.40%). Despite this, ALA is the first precursor in the metabolic pathway leading to EPA by desaturases and elongases^24^, and is an essential fatty acid for humans that just could obtain from food. Silkworm-based sources of *n*-3 PUFAs are easy to access, highly efficient, green and safe, which make them more advantageous and promising as a source of *n*-3 PUFAs. Thus, silkworm-containing *n*-3 PUFAs could be an ideal source for the supply of these fatty acids.

### Interesterification reaction

**Figure 2:**
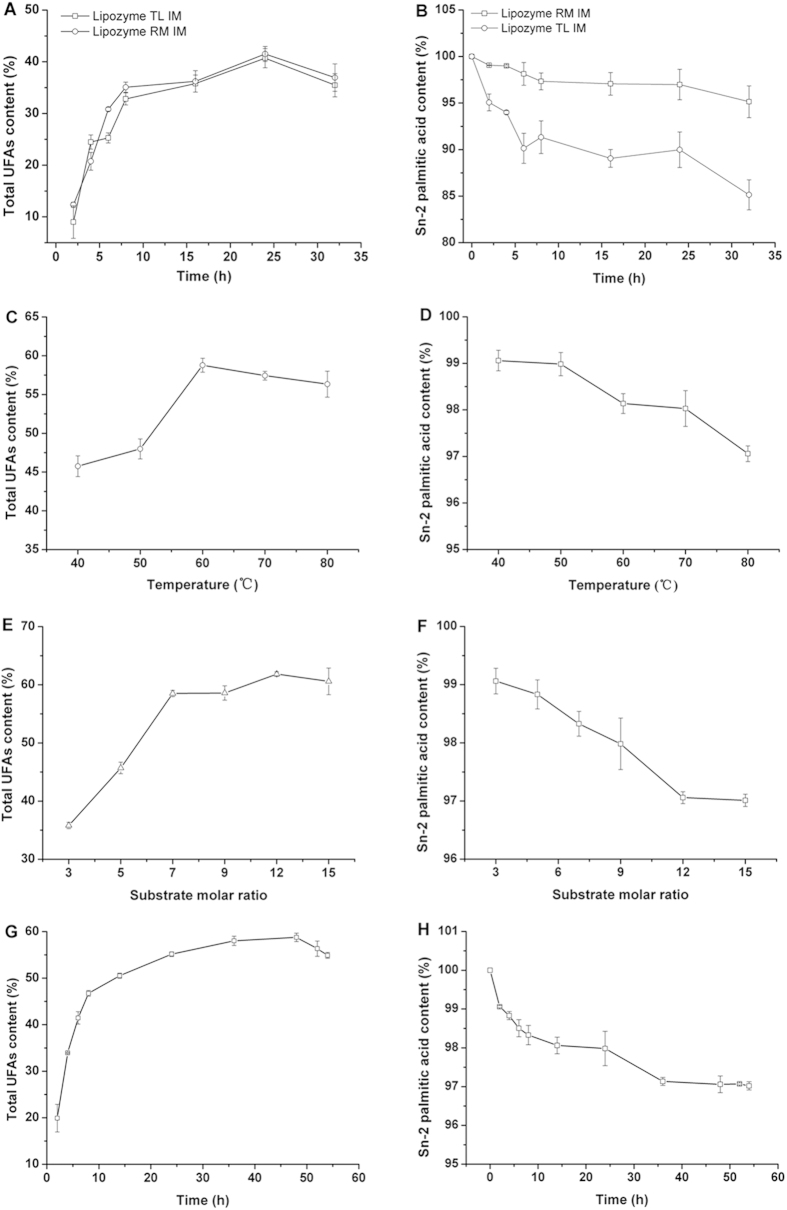
Effect of different conditions on the total content of ALA and palmitic acid in *sn*-2 position during the acidolysis of glyceryl tripalmitate with UFFA. Conditions: lipases (**A,B**), enzyme load = 10% (wt), molar ratio = 3:1, temperature = 60 °C, time = 32 h; reaction time (**C,D**), 10% (wt) of Lipozyme RM IM, molar ratio = 7:1, temperature = 60 °C; temperature (**E,F**), 10% (wt) of Lipozyme RM IM, molar ratio = 7:1, time = 48 h; molar ratio of FAs to PPP (**G,H**), 10% (wt) of Lipozyme RM IM, temperature = 60 °C, time = 48 h, respectively.

Figure 2 shows the effect of different conditions on the incorporation of UFAs during the acidolysis of PPP with UFFA from silkworm pupae oil. Enzymatic interesterification is often used to produce structured TAGs with the improved functionality by incorporating fresh FAs into the TAG. The *sn*-1, 3 specific lipases, Lipozyme RM IM and Lipozyme TL IM, were used to screen for optimum reaction conditions, and the results were showed in Fig. 2A. The lipase has more specific catalytic activity than other enzymes like Novozym 435 which usually used for interesterification. With the lipase, the UFAs were combined at *sn*-1, 3 more precisely. This figure showed that the initial rate of incorporation of UFAs in PPP was relatively higher with both lipases tested, but the maximum incorporation rates were obtained with Lipozyme RM IM (41.52%). Figure 2B shows the PA content at the *sn*-2 position of APA. This work aimed to incorporate UFAs at *sn*-1, 3 positions and maintain high PA incorporation at the *sn*-2 position. It was observed that the selectivity of Lipozyme RM IM was remarkably higher than Lipozyme TL IM, This is in consistence with previous reports^25^. Therefore, Lipozyme RM IM was selected for the catalyst in our investigation.

Figure 2C shows the effect of reaction temperature on the incorporation of UFAs during acidolysis of the PPP with UFFA. When the reaction temperature was below 60 °C, the total UFAs content increased as the temperature increased at the same substrate concentration. However, when the temperature increased to 80 °C, the total UFAs content significantly decreased. In general, the temperature of an enzymatic reaction is chosen according to the activity and stability of the enzyme, but the fact that PPP has a melting point of 66 °C must be also considered in this reaction. The substrates rich in PPP must remain in liquid form at the chosen temperature chosen, but the addition of organic solvent can reduce this temperature. Theoretically, an increase of reaction temperature results in an acceleration of the reaction based on the Arrhenius law. However, lipase will be deactivated at high temperature resulting to lower reaction rate^26^. Also, as shown in Fig. 2D, higher temperatures increases the rate of acyl migration, which may reduce the *sn*-1, 3 specificity of Lipozyme RM IM. Therefore, in the *n*-hexane system, 40, 50, 60, 70 and 80 °C were chosen as the experimental temperature, and the result shows 60 °C as the best reaction temperature.

Figure 2E shows the effect of the substrate molar ratio on the incorporation of UFAs during the acidolysis of PPP with UFFA. When the substrate molar ratio of UFFA to PPP was set at 12:1, the content of ALA was 61.85%, which was significantly different from the other reaction conditions. However, when the substrate molar ratio of UFFA to PPP rose to 15:1, the total UFAs content was essentially unchanged. Provided that the lipase shows the same selectivity toward all FAs and also the same regiospecificity toward *sn*-1 and *sn*-3 positions, the theoretical equilibrium or maximum incorporation of all acyl donors can be calculated using the following equation, if no side reactions occur:


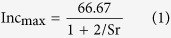


Where Inc_max_ is the equilibrium acyl incorporation and S_r_ is the substrate mole ratio of FFA to PPP.

In this study, the effect of acyl donor substrate concentration on the incorporation of UFAs was investigated. Theoretically, when the S_r_ is 3, 5 and 7, Inc_max_ is 39.92, 47.62 and 51.88, respectively. However, when S_r_ increased from 3 to 7, ALA incorporation also increased (Fig. 2E). The results are also consistent with Eq. (1) with respect to maximum incorporation. Under the conditions of high-substrate ratios, substrate inhibition, enzyme saturation or partial loss of lipase activity may occur. High levels of FFA could produce high levels of free or ionized carboxylic acid groups, which would acidify the micro aqueous phase surrounding the lipase or cause adsorptions of water from the interface. Also, higher FFA may increase the rate of acyl migration (Fig. 2F). However, in this interesterification reaction, more UFAs were added into the *sn*-2 position rather than being introduced into the *sn*-1, 3 positiondue to the acyl migration effect. Acyl migration is complex and could not be completely avoided, but it can be minimized^27^. Compared to solvent-free systems, solvent systems are much more stable, because of the increased solubility and mobility of dissolved substrate^28^. A high rate of incorporation and low rate of acyl migration for Lipozyme RM IM were observed under these reaction conditions. Therefore, the possible reason for the decrease in incorporation of ALA may be ascribed to inhibition of Lipozyme RM IM at higher substrate (FFA) concentrations, and therefore the substrate molar ratio of UFFA to PPP at 12:1 was used for future investigation.

Figure 2G shows the effect of reaction time on the incorporation of UFAs during the acidolysis of PPP with UFFA. The UFAs content increased with the reaction time. In the first 8 h, reaction rate is relatively high. However, the reaction rate began to decrease during the 8^th^ h to 36^th^ h. From 36 h to 48 h, the rate was practically constant. The incorporation of UFAs increased slowly until 48 h with highest content of UFAs of 58.78%. In addition, high PA content of 97.07% in the *sn*-2 position was decreased all the time in the transesterification reaction (Fig. 2H).

Overall, two widely used immobilized *sn*-1, 3 regiospecific lipases Lipozyme RM IM and Lipozyme TL IM perform distinctly in different solvent systems. The best results were achieved with 10% (total weight) Lipozyme RM IM in *n*-hexane with a substrate molar ratio of 12:1 (UFAs: PPP) at 60 °C. After a 48 h reaction, 97.07% PA at the *sn*-2 position and 64.52% UFAs in the APA- product were obtained. In addition, the total content of ALA in the product reached 48.59%, which contains with 70.93% *sn*-1, 3 APA-style HMFA. Most of the ALA (96.22%) was esterified in the *sn*-1, 3 positions, which could be used as nutrition intensifying agent.

Compared with the previous work in our lab (Table 2), the APA-style HMFA provided essential fatty acids in the *sn*-1, 3 positions of the TAG and contained high content of PA in the *sn*-2 position^29^. The results reveals that APA-style HMFA has the most PA at *sn*-2 and ALA at *sn*-1, 3 positions, which shows higher reaction efficiency compared to others like algae oil SLs and olive oil SLs. Several studies have pointed to fat digestion and absorption of dietary TAG to explain the efficient absorption of PA when located at the *sn*-2 position^30^,^31^. It has been suggested that pancreatic lipase attack the TAGs in the intestinal lumen with a high degree of positional specificity. Lipolysis occurs predominantly at the *sn*-1 and *sn*-3 positions, and has showed to transform a 2-monoglyceride (2-MG) and two FFAs. The 2-MGs formed efficiently, diffused into the enterocytes from micelles and well absorbed^32^. This is the major reason why HMF is well absorbed, and provides 50% of the dietary energy requirements for the infant. Other sources of similar FA composition (e.g. plant oil, lard) contained the saturated FA esterified at the *sn*-1, 3 positions. Consequently, the FA composition and the distribution of dietary TAG have been the targets in infant formula studies.

### Model fitting

**Figure 3:**
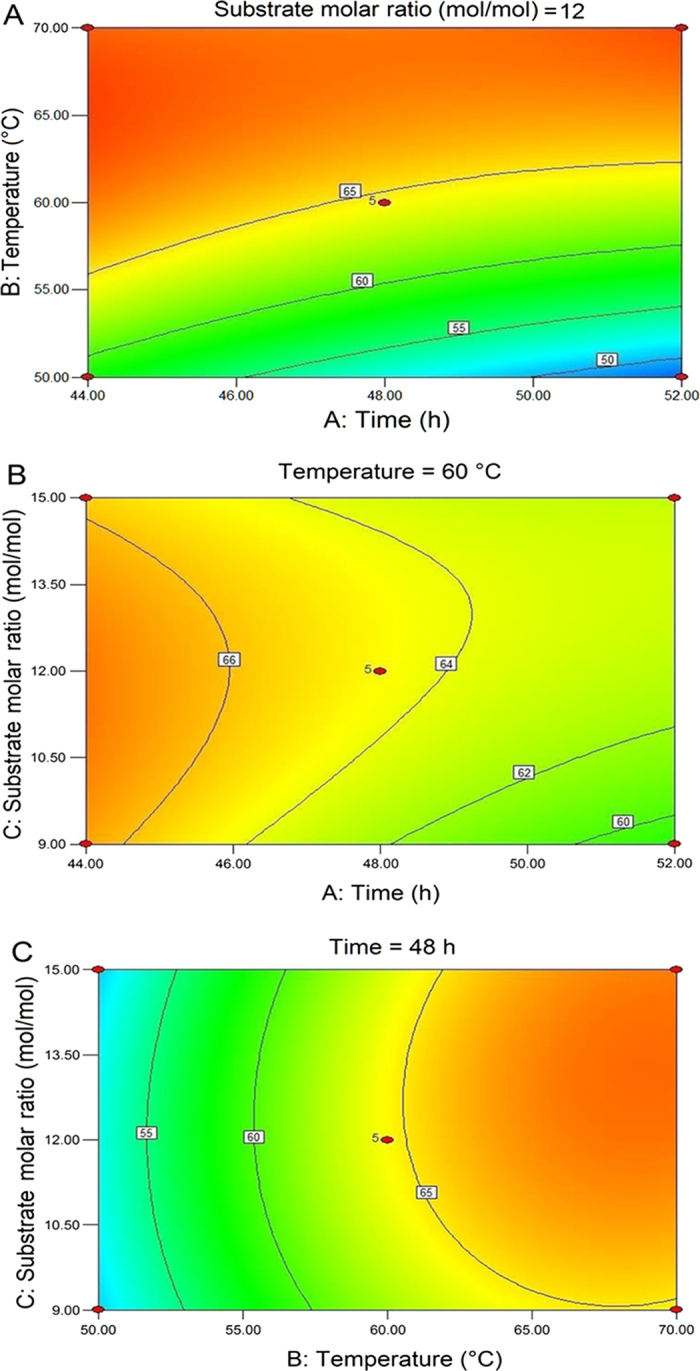
Contour plots of two parameters relating to UFAs incorporation: (**A**) reaction time versus temperature; (**B**) reaction time versus substrate molar ratio (mol/mol); (**C**) reaction temperature versus substrate molar ratio (mol/mol).

Response surface applications is an effective method to analyze the effect of many factors at the same time. Figure 3 shows the contour plots comparing two parameters for UFAs incorporation. The effects of: time (44, 48 and 52 h) (Fig. 3A), reaction temperature (50, 60 and 70 °C) (Fig. 3B) and substrate mole ratio of UFFA fraction to PPP (9:1, 12:1 and 15:1) (Fig. 3C) on UFAs content (Y) were considered in the present study. Table 3 shows the observed responses (Y) from each of the 17 experimental conditions generated by response surface methodology (RSM). Multiple linear regression (MLR) was used to fit a linear equation for a response using reaction factors. To provide the best fit to the model, a backward-elimination procedure was applied to remove variables with higher *P*-values (*P* > 0.05) that not make a significant contribution to a prediction. Table 4 displays the analysis of variance (ANOVA) of response surface for the model of UFAs content, showed that the model is significant (*P* value < 0.05); there is only a 3.06% chance that a “Model F-Value” this large could occur due to noise, and the lack-of-fit was not significant (*P* = 0.117). The R^2^ values (the fraction of variation of the response explained by the model) and R^2^_Adj_ (the fraction of variation of the response that can be predicted by the model) for measuring the goodness of the fit were 0.852 and 0.661, respectively^33^. The values were closed to 1 for both R^2^ and R^2^_Adj_ indicated a good model with excellent predictive power. Thus, the statistical values suggest that the response surface models for ALA content were adequate and well fitted. Regression coefficients and significance (*P*) values of the generated models for ALA acid incorporation are given in Table 5, and the predictive response surface equations are:


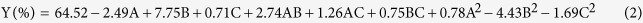


**Figure 4:**
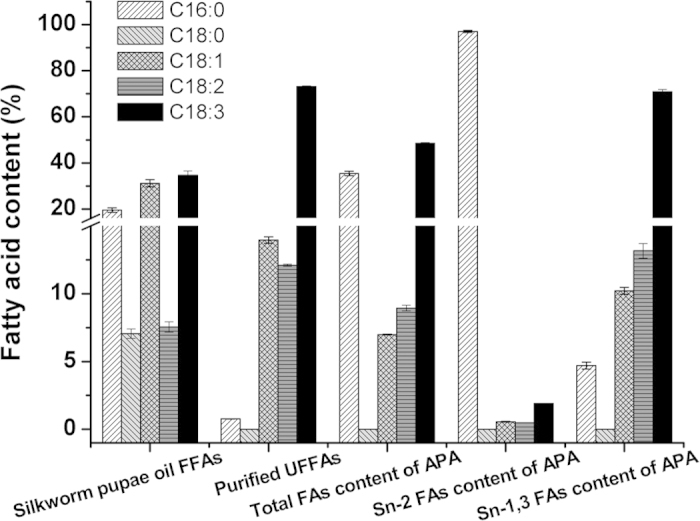
A comparison of the fatty acid content of free fatty acids from silkworm pupae oil (Silkworm pupae oil FFAs), purified UFFAs after the urea inclusion method from crude silkworm pupae oil (Purified UFFAs), total FA content of APA-style HMFA from silkworm pupae oil (Total FAs content of APA), *sn*-2 fatty acid content of APA-style human milk fat analogue from silkworm pupae oil (*sn*-2 FAs content of APA) and *sn*-1, 3 fatty acid content of APA-style HMFA from silkworm pupae oil (*sn*-1, 3 FAs content of APA).

According to the predictive response surface equations, the optimum reaction conditions are as follows: 48 h reaction time, 60 °C reaction temperature and a substrate mole ratio of UFFA to PPP of 12:1. The predicted UFAs incorporation was 64.52%, which was consistent with experimental verification. Under optimal reaction conditions, all the fatty acids were identified by GC analysis. Table 2 shows the FA composition of APA-style structured lipids from silkworm pupae oil under the optimum conditions, with a comparison of this product with other structured lipids (SLs). The major FAs in APA-SLs were ALA (48.59%), palmitic acid (35.48%), linoleic acid (8.94%) and oleic acid (6.99%). Compared with our previous study^29^, silkworm pupae oil SLs (SPOSLs), with low PA and high ALA, oleic acid (OA) and linoleic acid (LA) contents were synthesized from SPO using a solvent-free system catalyzed by Lipozyme RM IM. The APA-SLs maintained higher ALA content than SPOSLs. For the *sn*-2 FAs, 97.05% of PA content in the *sn*-2 position had been obtained. Figure 4 also compared the fatty acid content of FFA from silkworm pupae oil (Silkworm pupae oil FFA), purified UFFA after the urea inclusion method from crude silkworm pupae oil (Purified UFFA), total FA content of APA-style HMFA from silkworm pupae oil (Total FA content of APA) and *sn*-2 fatty acid content of APA-style HMFA from silkworm pupae oil (*sn*-2 FA content of APA). These structured TAGs are rich in *sn*-2 PA and containing high ALA at *sn*-1, 3 positions, which is the principal TAG of HMF, and these APA-style TAGs can be used to achieve nutritional fortification for infants.

### Reuse of lipase

**Figure 5:**
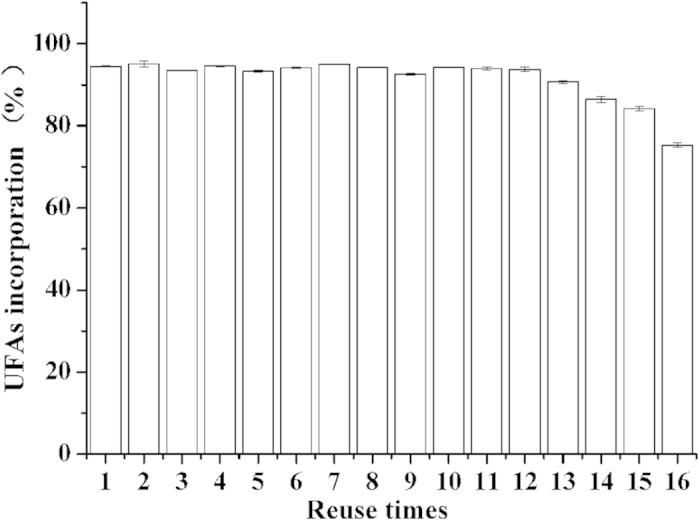
Reusability and service life of Lipozyme RM IM in continuous flow transesterification synthesis of SLs. Reaction conditions: reaction time: 48 h, molar ratio: 1:12, and temperature: 60 °C.

The reusability of the Lipozyme RM IM was studied with the optimal condition. Figure 5 shows the enzyme can be used for 16 times (32 d) with the activity of that is beyond 80%. The results show that the catalytic stability of the Lipozyme RM IM is so high that can efficiently decrease the cost of reaction.

### Melting and crystallization profiles

**Figure 6:**
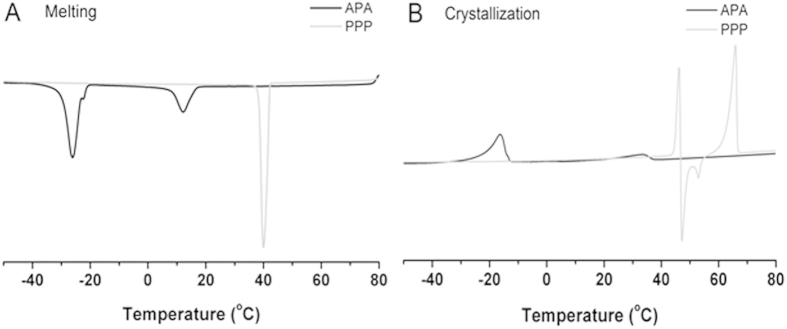
Melting (**A**) and crystallization (**B**) profiles of APA and PPP products.

Figures 6A,B show the melting and crystallization profiles of product APA-style TAGs and PPP. The melting and crystallization profiles of the fats were closely correlated with their chemical compositions. The melting curves are important for investigating physical state of the fat within the human body. Only fat with a melting point below the physiological temperature (36.6-37.3 °C)^34^, can be quickly emulsified and absorbed. The melting and crystallization curves of APA-style TAGs were lower than the physiological temperature. Compared with PPP (40.09 °C), the majority of the components in the structured TAGs melted in the middle of the melting point (− 26.45 °C), which may indicate that LC-PUFAs was dispersed in the structured TAGs products. In addition, the crystallization properties of the fat are of great importance for application. High initial milk fat crystallization temperatures were correlated with high final melting temperatures and vice versa. Reaction products showed the highest starting crystallization temperatures of −12.12 °C, with a corresponding highest final melting temperature of −20.12 °C of these APA-style TAGs. The same as the previous discussion, fat with a lower melting point than the physiological temperature (36.6–37.3 °C) can be emulsified and absorbed quickly. After the reaction, the thermograms peaks of structured TAGs shifted to the right and broadened indicating the improved plasticity and storage range of structured TAGs.

### Antioxidant activity of alkyl caffeates

**Figure 7:**
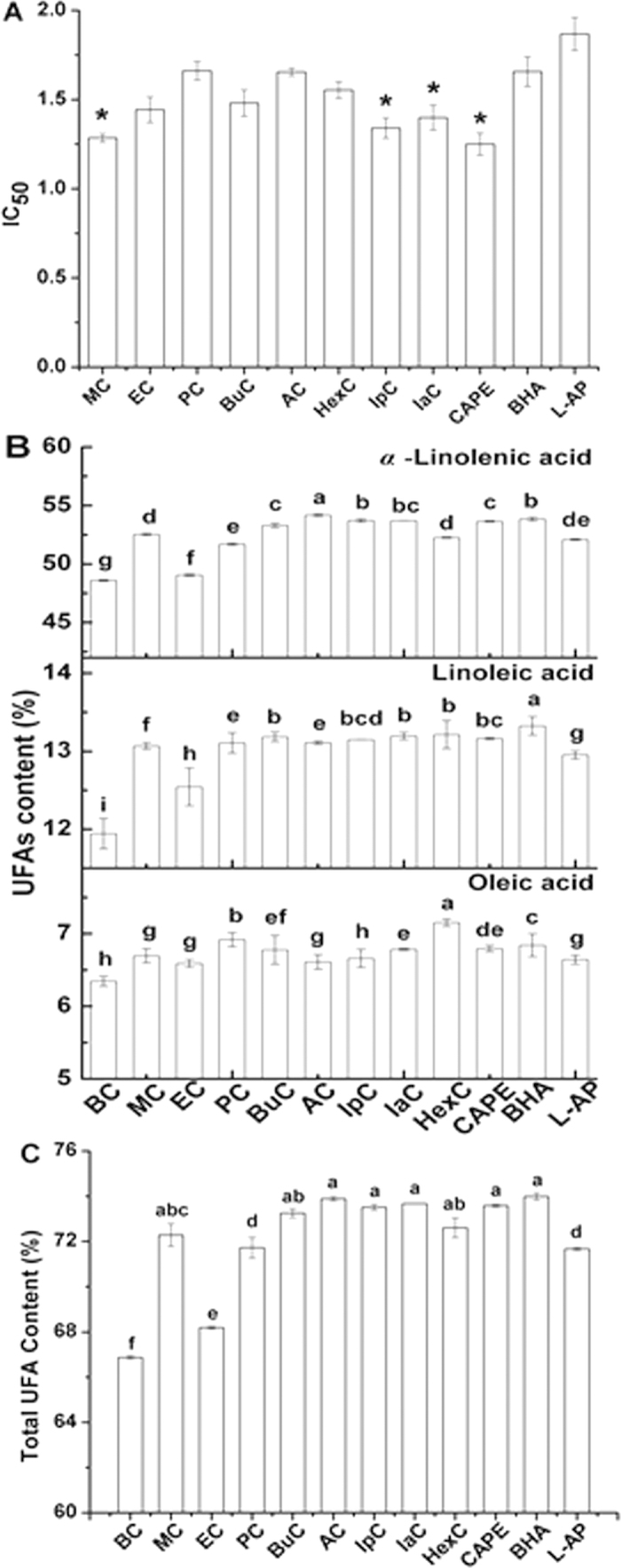
Effect of different antioxidants on APA-style SLs. (**A**) DPPH radical-scavenging activity of different antioxidants. Data are shown as IC_50_ (l *μ*g/ml). Results are the mean of three separate determinations ± SEM. *P* < 0.05 vs BHA and L-AP. (**B**) The contents of the different UFAs by adding different antioxidants, different letters indicate significant differences (*P* < 0.05) from BC. (**C**) The total content of UFAs by adding different antioxidant, different letters indicate significant differences (*P* < 0.05) from BC.

Figure 7A shows the half-inhibitory concentration (IC_50_) for the radical-scavenging activity of CAPE and alkyl caffeates with different chain lengths. The traditional antioxidants for oil and fat, BHA and L-AP, were used for comparison. The inhibition ratio of alkyl caffeates for DPPH radical-scavenging was as follows: CAPE > MC > IpC > IaC > EC > BuC > HexC > AC > PC > BHA > L-AP. This result indicated that the alkyl caffeates were potent antioxidants because a higher DPPH scavenging ability was obtained, compared to commercial BHA and L-AP fat-soluble antioxidants. The IC_50_ values for CAPE and MC were 1.25 and 1.29 *μ*g/ml, respectively, while the IC_50_ values of BHA and L-AP were 1.66 and 1.87 *μ*g/ml, respectively. Thus, CAPE and MC were more efficient antioxidants than BHA and L-AP. In addition, IpC, IaC, EC, BuC, HexC, AC and PC showed more significant antioxidant activity than BHA and L-AP (*P* < 0.05). Among these antioxidants, CAPE was the most efficient in eliminating free radicals in this product, which agreed with earlier studies. Therefore, the alkyl caffeates esterified from CA and the alkyl alcohols, especially CAPE, can prevent structured TAGs oxidation during the processing and storage period. In a practical application, alkyl caffeates esterificated from CA and the alkyl alcohols could completely replace BHA and L-AP, especially in infant formula.

APA-style TAGs contain a large quantity of UFAs (64.52%), which can effectively prevent a variety of diseases. Unfortunately, these UFAs are liable to be oxidized. Increasing attention has been attracted to stabilize edible oil, especially for its storage, to improve the quality and safety of the oil. The resistance to oxidants is assessed by several factors: antioxidant type, antioxidant amount, storage conditions and oil fatty acid profile, especially the UFAs content^35^. The oxidative products degraded the quality of oil, shorten its storage time and threaten human health. Higher antioxidant activity is necessary within structured TAG products to inhibit oxidation. This is essential in maintaining product safety, especially for infants. Therefore, more attention should be focused on the development of natural sources of liposoluble antioxidants. In the present study, alkyl caffeates were investigated as antioxidants to demonstrate their practical value with our APA-style HMFA.

Figure 7B shows the UFAs (C18:1, C18:2 and C18:3) content of APA-STAGs with the addition of alkyl caffeates, BHA and L-AP or no antioxidants addition (BC: blank control) at 180 °C. Overall, the content of UFAs increased with the addition of alkyl caffeates, BHA and L-AP. For the total content of unsaturated fatty acids in APA-style TAGs (Fig. 7C), the highest effective antioxidant was AC at 180 °C for 4.5 h. However, the antioxidant effect of some antioxidants, such as BuC, IpC, IaC and CAPE was not significantly different from AC (*P* < 0.05), which was shown in Table 6, suggesting that the natural sources of antioxidants could improve the storage ability of STAGs.

In conclusion, APA-style HMFA was successfully produced by silkworm pupae oil. The structured TAGs with 94.31% of UFAs at the *sn*-1, 3 positions and the palmitic acid of 97.05% were obtained via the interestrification. The result shows the higher reaction efficiency that other similar work like the olive oil SLs recently cannot reach^12^. The ALA content in the modified oil reached 48.59%. Characterization showed that APA-style HMFA with improved plasticity and storage ranges may be used for nutritional enhancement. In addition, alkyl caffeates demonstrated obvious antioxidant activity on APA-style HMFA. Despite the advancements of our technique, the cost of enzymes and the time-consuming process limited the industrial utilization of this process. Future work will focus on the increase of the enzymatic efficiency and reduce the cost of this process to assure an economic industrial application.

## Methods

### Preparation of free fatty acids (FFAs) from the silkworm pupae

**Figure 8:**
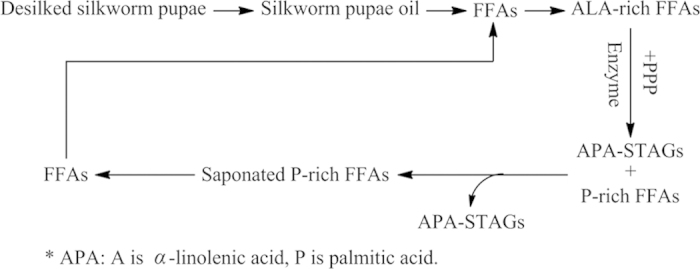
From FFAs preparation to APA-style HMFA production via enzymatic transesterfication using desilked silkworm pupae as feedstock.

The desilked silkworm pupae were dried at 60 °C in a DHG-9240A drying oven (Shanghai Yiheng Scientific instruments, Shanghai, China), powered by an herb disintegrator (Qinzhou Sanyang Package Equipment, Qinzhou, China) and then sieved (60 meshes). The oil was extracted from the silkworm pupae powder using ultrasonic-assisted (60 watts) solvent extraction (*n*-hexane). The offscourings were removed by filtration, and the oil-*n*-hexane mixture was subjected to vacuum rotatory evaporation until no *n*-hexane remained. The crude desilked silkworm pupae oil (500 g) was then saponified by refluxing it for 6  h at 65 °C under a nitrogen atmosphere using a mixture of NaOH (8.0  g) and 95% v/v aqueous ethanol (2 L). This was performed until the whole mixture became a clear, homogeneous solution. The remaining ethanol was recovered under reduced pressure. To dissolve the saponified mixture, distilled water (500 mL) was added and the aqueous layer, containing saponified material, was acidified (pH 3-4) with 10% HCl. The solution was shaken and held for 1 h, and the water layer was removed. Petroleum ether (boiling point 60–90 °C, 500 mL) was added to extract the oil layer. Distilled water (500 mL, 3 times) was added to wash the oil layer until it was neutral. The petroleum ether layer, containing free fatty acids, was then dried over anhydrous magnesium sulfate and the solvent was removed at 50 °C to recover crude FFAs (300 g). Thus, from FFAs preparation to APA-style HMFA production via enzymatic transesterfication using desilked silkworm pupae as feedstock, which whole process was shown in Fig. 8.

### Enrichment of ALA from crude FFAs

The separation of ALA from FFAs was carried out by urea complexation^11^,^36^. Crude FFAs extracted from the desilked silkworm pupae oil were dissolved in ethanol, and then slowly added to a separatory funnel over 10–15 min. The rest of the oil was washed by 95% of ethanol. The ratio of saturated urea/EtOH solution to FFAs was 2:1 (v/v). The mixture was stirred at 4 °C for 2 h until the solution was clear under a slow flow of nitrogen. The mixture was transferred into conical flasks when the reaction was completed.

### Interesterification Reaction

The mixtures of reactants (PPP and FFAs) that were prepared at different substrate mass ratios were placed in a 50 mL conical flask and treated with enzymes in a 220 rpm orbital shaking water bath. Lipozyme RM IM and Lipozyme TL IM were tested in this reaction. The amount of PPP (0.1 mmol) was held constant, but the amount of unsaturated FFAs (UFAs) varied (0.3–1.5 mmol). The molar ratios 1:3, 1:5, 1:7, 1:9, 1:12 and 1:15 were set to achieve the desired FFA to TAG molar ratio. This mixture was added with 2 mL of *n*-hexane. The reaction temperatures of 40, 50, 60, 70 and 80 °C were tested. The enzyme loading was set at 10% by weight of total substrates. The reaction time was maintained for 54 h, and samples were withdrawn at 2, 4, 6, 8, 14, 24, 36, 48, 52 and 54 h. Samples were prepared in duplicate and stored in a freezer prior to analysis.

To remove FFAs in the final products, 50 mL of hexane and 10 drops of phenolphthalein solution were added and the final products were titrated with 0.5 M KOH in 95% ethanol until a pink color appeared. The reaction mixture was washed several times with water until the pink color disappeared. The saponated FFAs were recovered and acidified again, to enrich for the ALA which had not participated in prior reaction. The organic layer was passed through an anhydrous sodium sulfate column to remove moisture, and the solvent was completely evaporated under nitrogen at 40 °C.

### Experimental design for response surface method

A three-factor, rotatable three level BBD was employed to generate factor combinations by using Design Expert 8.05 b (Stat-Ease) software. The three factors chosen were substrate molar ratio (Sr, UFFA/PPP, 9–15 mol/mol), temperature (T, °C, 50–70 °C), and reaction time (t, hours, 44–52 h). A total number of 17 runs were generated by the software. The independent variables and experimental design are shown in Table 3. Experiments were run randomly, and duplicate reactions were carried out at all design points.

### Fatty acid composition and positional profiles

The fatty acids of substrates and reaction products were converted into fatty acid methyl esters (FAMEs) according to the procedure described by O. Fallon and others^37^. The positional analysis of structured TAGs was performed using the pancreatic lipase-catalyzed *sn*-2 method^38^. FAMEs were analyzed using gas chromatography (GC) and the FA content and relative percentage of FA at the *sn*-2 position in the synthesized TAGs was calculated as follows:


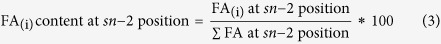



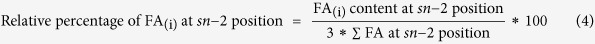


From the total FA content of a TAG and the content of this FA at the *sn*-2 position, the content at the *sn*-1, 3 positions may be calculated using the following equation:


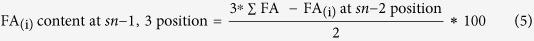


Where FA_(i)_ represents different fatty acids (when i = 1, 2, 3, 4, 5, it represents C16:0, C18:0, C18:1, C18:2 and ALA respectively); ∑ FA is the total amount of fatty acids.

### GC analysis and GC-MS

The FA composition of the FAMEs was determined via capillary GC on a hp-innowax, 30 m × 0.25-mm id, 0.25-*μ*m capillary column (Model 6820N; Agilent) installed on an Agilent Technologies gas chromatograph equipped with a Hewlett Packard 3396 Series II integrator and 7673 controller, a flame ionization detector, QA/QC nds cerity system and split injector (Agilent Technologies Inc., Santa Clara, CA). The gas chromatographic conditions were as described by Wang *et al.* but were revised^39^. The initial oven temperature was 200 °C, and held for 1  min, and then the temperature was increased to 220 °C at a rate of 1.5 °C/min. The column head pressure was 280 kPa. The injector was set to 250 °C and the detector to 280 °C. The split ratio was 50:1; the carrier gas was nitrogen with a flow rate of 1mL/min. FAs were identified by comparing their retention times with FAME standards.

GC-MS analysis was performed using an Agilent 6890 gas chromatograph with a 5973 MS detector equipped with 60 m × 0.25-mm i.d. 0.25-μm/MS capillary column (DB-5). The following temperature program was used: the injector was set at 250 °C, the oven was initially set at 200 °C, held for 1 min and then heated to 230 °C (1.5 °C/min, then held for 10 min). The characterization and identification of FAMEs was completed in scan mode^40^. The selected ion-monitoring mode was from 35 to 320 m/z.

### Melting and crystallization profile

Melting and crystallization profiles were determined for structured TAGs and PPP using a differential scanning calorimeter (DSC model DSC7, Perkin-Elmer Co., Norwalk, CT). The method of Lee and others was used^41^, with minor modifications: indium and *n*-decane were used as standards. Samples were weighed in aluminum pans and ranged from 8 to 12 mg. To destroy any previous crystalline structures, samples were heated from 25 to 80 °C at 50 °C /min and held for 10 min. For crystallization profiles samples were then cooled from 80 to 55 °C at 10 °C/min and held for 30 min. To get melting profiles the samples were then heated from 55 to 80 °C at 5 °C/min. Dry ice and acetone were used as the coolants. The thermograms were analyzed using DSC (Pyris software, Perkin-Elmer, Shelton, CT).

### DPPH Assay

The DPPH-scavenging assay was performed to elucidate the radical-scavenging properties of CA and its alkyl derivatives^42^. An ethanol solution of DPPH is purple and has an absorption maximum at 517 nm^43^. In the presence of antioxidants, DPPH captures an electron causing the molecule to lose its absorbance at 517 nm^44^. The alkyl caffeates were dissolved using ethanol, and different concentrations were mixed with a methanolic solution of DPPH. The mixtures were then incubated for 30 min in the dark at room temperature. The 96 well micro-plates were read in a SpectraMax i3 Multi-mode detection platform (Molecular Devices Co., San Francisco, US).

### Effect of different antioxidants

Eight alkyl caffeates, CAPE, BHA and L-AP were added to the product structured TAGs. The antioxidant concentrations were set at 0.02% (molar ratio). The samples were heated in an oil bath at 180 °C for 5 h. After the reactions, 0.2 g sample was dissolved with 0.5 mL of n-hexane in a 10 mL tube. 2 mol/L KOH-CH_3_OH solution (0.2 mL) was added to the tube^18^. The structured TAGs were transformed into FAMEs, and the FAME content was determined using the GC methods described above.

### Statistical analysis

All experiments were performed in triplicate and the standard deviation was calculated to check the reliability of the results. Statistical analysis was performed using ANOVA. Statistical comparison between data was based on the Pearson correlation coefficient, with *P* < 0.05 considered significant. Statistical analysis was performed using SPSS 19.0, and response surface applications were performed using Design-Expert 8.05 b (Stat-Ease, Inc., US). Duncan’s multiple range tests were performed to determine the significance of difference at *P* < 0.05.

## Additional Information

**How to cite this article**: Liu, X. *et al.* APA-style human milk fat analogue from silkworm pupae oil: Enzymatic production and improving storage stability using alkyl caffeates. *Sci. Rep.* **5**, 17909; doi: 10.1038/srep17909 (2015).

## Supplementary Material

Supplementary Information

## Figures and Tables

**Figure 1:**
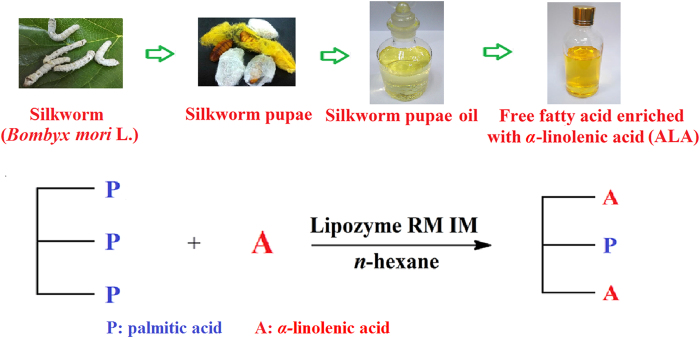
Biosynthesis diagram of APA-style HMFAs from silkworm pupae oil via enzymatic transesterification. The photos were taken and modified by X. L., and the diagram was drawn by X. W.

**Table 1:** Comparison of FFA content before and after the urea inclusion method from crude silkworm pupae oil.

Fatty acid	Content (%)		
	Silkworm pupae oil	Urea inclusion	ALA concentrate^d^
Palmitic acid (C16:0)	19.50 ± 0.97	0.77 ± 0.01	12.60
Stearic acid (C18:0)	7.05 ± 0.35	ND^c^	ND^c^
Oleic acid (C18:1 *n*-9)	31.16 ± 1.56	13.96 ± 0.24	13.60
Linoleic acid (C18:2 *n*-6)	7.55 ± 0.38	12.10 ± 0.08	6.40
Linolenic acid (C18:3 *n*-3)	34.73 ± 1.74	73.16 ± 0.30	67.40

^a^Mean ± SD, n = 3. ^b^Content less than 0.5 was not displayed. ^c^ Not detected. ^d^ adapted from Wang *et al.*, 2013.

**Table 2:** FA composition of APA-style structured lipids from silkworm pupae oil under the optimum conditions, and comparison of this product with other SLs.

Fatty acid	C8:0	C12:0	C16:0	C18:	C18:1	C18:2 *n*-6	C18:3 *n*-3	C22:5 *n*-6	C22:6 *n*-3
Total fatty acid content (%)									
APA-SLs^a^	—	—	35.48	—	6.99	8.94	48.59	—	—
SPOSLs^b^	—	—	11.85	3.86	51.61	23.73	12.79	—	—
AOSLs^c^	—	—	9.51	1.33	27.82	11.86	—	12.63	30.11
Olive oil SLs^d^	—	—	55.79	1.01	36.54	3.37	—		3.01
Tuna oil/CA^e^	45.2	—	12.40	2.00	7.20	—	—	4.70	16.20
*sn*-2 fatty acid content (%)									
APA-SLs^a^	—	—	97.05	—	0.56	0.48	1.91	—	—
SPOSLs^b^	—	—	3.59	2.22	42.06	9.30	42.38	—	—
AOSLs^c^	—	—	13.64	3.34	27.83	11.86	—	12.63	30.11
Olive oil SLs^d^	—	—	33.63	—	66.37	—	—	—	—
Tuna oil/CA^e^	12.81	—	22.00	1.50	8.80	—	—	7.80	24.90
*sn*-1, 3 fatty acid content (%)									
APA-SLs^a^	—	—	4.70	—	10.21	13.17	70.93	—	—
SPOSLs^b^	—	—	16.43	3.68	48.09	28.30	1.49	—	—
AOSLs^c^	—	—	6.95	0.32	21.98	13.24	—	15.52	35.88
Olive oil SLs^d^	—	—	—	—	—	—	—	—	—
Tuna oil/CA^e^	61.40	—	7.60	2.30	6.40	—	—	3.20	11.90

Not all fatty acids are listed in the table above, this only lists the differences with the part of silkworm pupae oil interesterified product SLs.

^a^FA composition of APA-style structured lipids from silkworm pupae oil under the optimum conditions.

^b^adapted from Zhao *et al.*, 2014.

^c^adapted from Wang *et al.*, 2014.

^d^adapted from Li *et al.*, 2014.

^e^adapted from Teichert, 2011.

**Table 3:** Results of Box-Bohnkon on synthesis of APA-style structured lipids from silkworm pupae oil.

Std.	Run	Time (h)	Temperature (°C)	Molar ratio of UFFA to PPP	UFAs Incorporation (%)	
					Observed	Predicted
1	4	44.00	50.00	12:1	61.63	58.35
2	11	52.00	50.00	12:1	45.49	47.90
3	5	44.00	70.00	12:1	70.78	68.37
4	12	52.00	70.00	12:1	65.59	68.87
5	8	44.00	60.00	9:1	65.40	66.66
6	7	52.00	60.00	9:1	63.58	59.15
7	14	44.00	60.00	15:1	61.12	65.55
8	13	52.00	60.00	15:1	64.36	63.10
9	2	48.00	50.00	9:1	48.68	50.69
10	10	48.00	70.00	9:1	63.54	64.70
11	1	48.00	50.00	15:1	51.77	50.62
12	16	48.00	70.00	15:1	69.62	67.61
13	15	48.00	60.00	12:1	64.00	64.52
14	9	48.00	60.00	12:1	61.14	64.52
15	17	48.00	60.00	12:1	66.95	64.52
16	6	48.00	60.00	12:1	67.69	64.52
17	3	48.00	60.00	12:1	62.81	64.52

**Table 4:** Analysis of variance of Box-Bohnkon experiment on the synthesis of APA-style structured lipids from silkworm pupae oil.

Source	Sum of squares	DF	Mean square	F-value	*P*-value
Model	671.26	9	74.58	4.47	0.0306
Residual	116.90	7	16.70		
Lack of fit	86.30	3	28.77	3.76	0.1167
Pure error	30.60	4	7.65		
Cor. total	788.16	16			
R^2^	0.8517				
R^2^_Adj_	0.6610				

**Table 5:** Regression coefficients and *P*-values of the models obtained for incorporation of APA.

Variables	Coefficients	*P*-value^a^
intercept	64.52	<0.0001
A	−2.49	0.0761
B	7.75	<0.0001
C	0.71	0.6387
A × B	2.74	0.2221
A × C	1.26	0.5553
B × C	0.75	0.7252
A^2^	0.78	0.7056
B^2^	−4.43	0.0254
C^2^	−1.69	0.4250

^a^*P* value, level of significance. See Table 1 for other abbreviations.

**Table 6:** Inoxidizability of APA-style structured lipids from silkworm pupae oil with different antioxidants.

Antioxidant	Fatty acid content (%)		
	C18:1	C18:2	C18:3
BC	^h^ 6.34 ± 0.07	^i^ 11.94 ± 0.20	^g^ 48.59 ± 1.67
MC	^g^ 6.69 ± 0.50	^f^ 13.07 ± 0.44	^d^ 52.52 ± 0.24
EC	^g^ 6.59 ± 0.05	^h^ 12.54 ± 0.24	^f^ 49.05 ± 1.81
PC	^b^ 6.92 ± 0.46	^e^ 13.11 ± 0.13	^e^ 51.69 ± 1.46
BC	^ef^ 6.78 ± 0.20	^b^ 13.18 ± 0.13	^c^ 53.29 ± 0.85
AC	^g^ 6.61 ± 0.10	^e^ 13.11 ± 0.02	^a^ 54.17 ± 1.08
IpC	^h^ 6.66 ± 0.13	^bcd^ 13.15 ± 0.00	^b^ 53.70 ± 0.56
IaC	^e^ 6.78 ± 0.01	^b^ 13.19 ± 0.05	^bc^ 53.70 ± 0.67
HexC	^a^ 7.15 ± 0.44	^b^ 13.21 ± 0.18	^d^ 52.24 ± 1.73
CAPE	^de^ 6.79 ± 0.05	^bc^ 13.16 ± 0.02	^c^ 53.63 ± 0.91
BHA	^c^ 6.84 ± 0.16	^a^ 13.32 ± 0.12	^b^ 53.83 ± 0.36
L-AP	^g^ 6.64 ± 0.06	^g^ 12.95 ± 0.05	^de^ 52.08 ± 0.15

The contents of the different UFA by adding different antioxidant, different letters indicate significant differences (*P* < 0.05) compared to BC.

a–i The mean values in the same row for the contents of different unsaturated fatty acid by adding different antioxidant are significantly different (*P* < 0.05).
