# Supplementary material for: APA-style human milk fat analogue from silkworm pupae oil: Enzymatic production and improving storage stability using alkyl caffeates
Source: Sci Rep. 2015 Dec 8;5:17909. doi: 10.1038/srep17909 (PMC4672268; doi:10.1038/srep17909)
Supplement: Supplementary Information [file srep17909-s1.doc]

**Supplementary materials for**

**APA-style human milk fat analogue from silkworm pupae oil: Enzymatic production and improving storage stability using alkyl caffeates**

Xi Liu, Xudong Wang, Na Pang, Weijie Zhu, Xingyu Zhao, Fangqin Wang, Fuan Wu, Jun Wang

**Methods**

*Determination of molar fractions of fatty acids in SL by GC and GC-MS*

Acylglycerols (monoacylglycerol and triacylglycerol) were identified by thin-layer chromatography (TLC) and the fatty acid composition of each acylglycerol type was carried out by gas chromatography (GC). Methyl Heptadecanoate (C17:1) (Sigma–Aldrich) was used as an internal standard for quantitative determination of fatty acids. The amounts of fatty acids were calculated by the equation (Hita et al., 2007):

(S1)

Where f_X_ is the fatty acid response factor of a determined fatty acid X. These response factors were close to 1 for all the fatty acids of molecular weight close to the internal standard, and therefore f_X_ = 1 was taken for all the fatty acids. These factors were calculated from Eq (1), by comparing chromatographic areas of known amounts of 17:0. All the chemicals used in this and in the following analytical methods described were of analytical quality and acquired from Sigma–Aldrich (St. Louis, MO).

***Results and discussion***

*Confirmation of products by GC and GC-MS*

Fig. S1 shows the GC-MS analysis of the FAMEs of PPP and APA-style SLs. For the purpose of clarity, the solvent peaks (*n*-hexane) are shown in Figure S1. The *n*-hexane front appears at a retention time of 1.17 min, followed by palmitic acid (C16:0), which appears at a retention time of 4.69 min (Fig. S1A). Fig. S1B was the mass spectrogram of n-hexane, C was methyl palmitate, E was *n*-hexane, F was methyl palmitate from APA and G was methyl linolenate. Because the methylation process converts FAs to FAMEs by derivatization using the KOH-methanol method, the main components are present in the PPP and APA-style SLs. All the FAME components were verified using MS.

**Supplementary Figure Legends:**

Fig.S1 The GC-MS analysis of the FAMEs of PPP and APA-style SLs. Typical GC-MS chromatogram changes of the FAMEs from PPP and APA prior to and after the reaction (A) and (D), and a representative mass spectrometry result of all species: (B) *n*-hexane (C) methyl palmitate, (E) *n*-hexane, (F) methyl palmitate, (G) methyl linolenate.


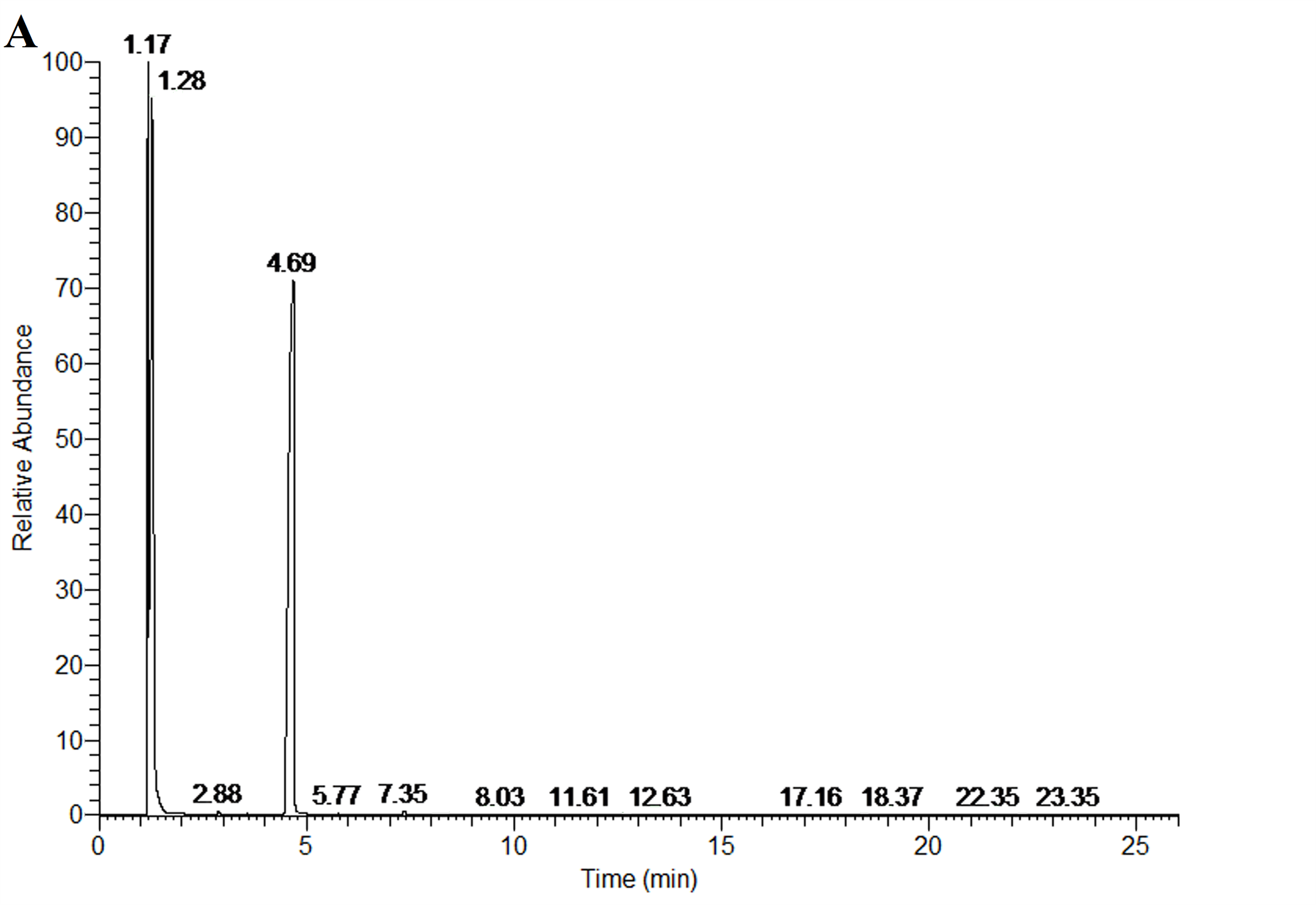


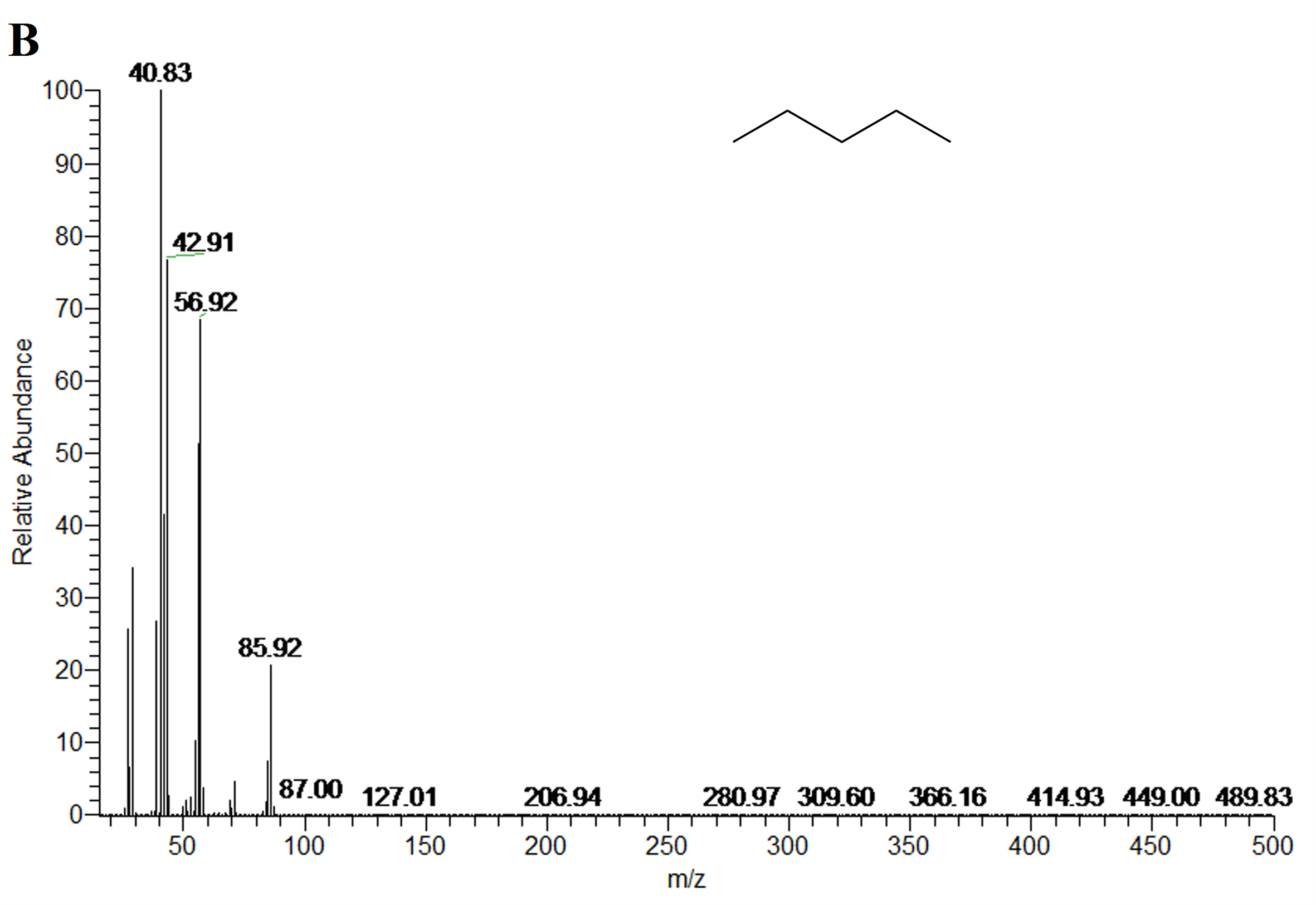


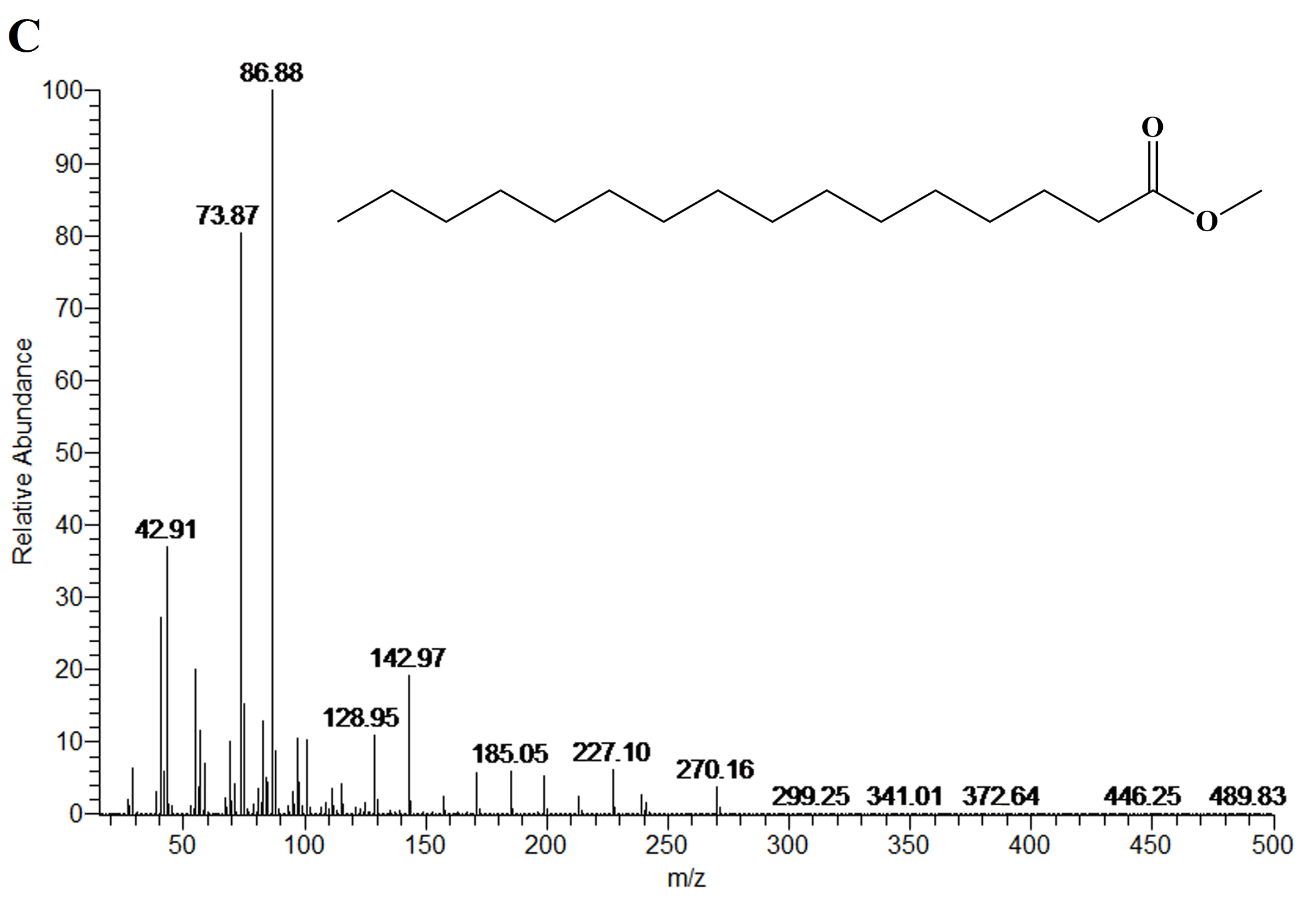


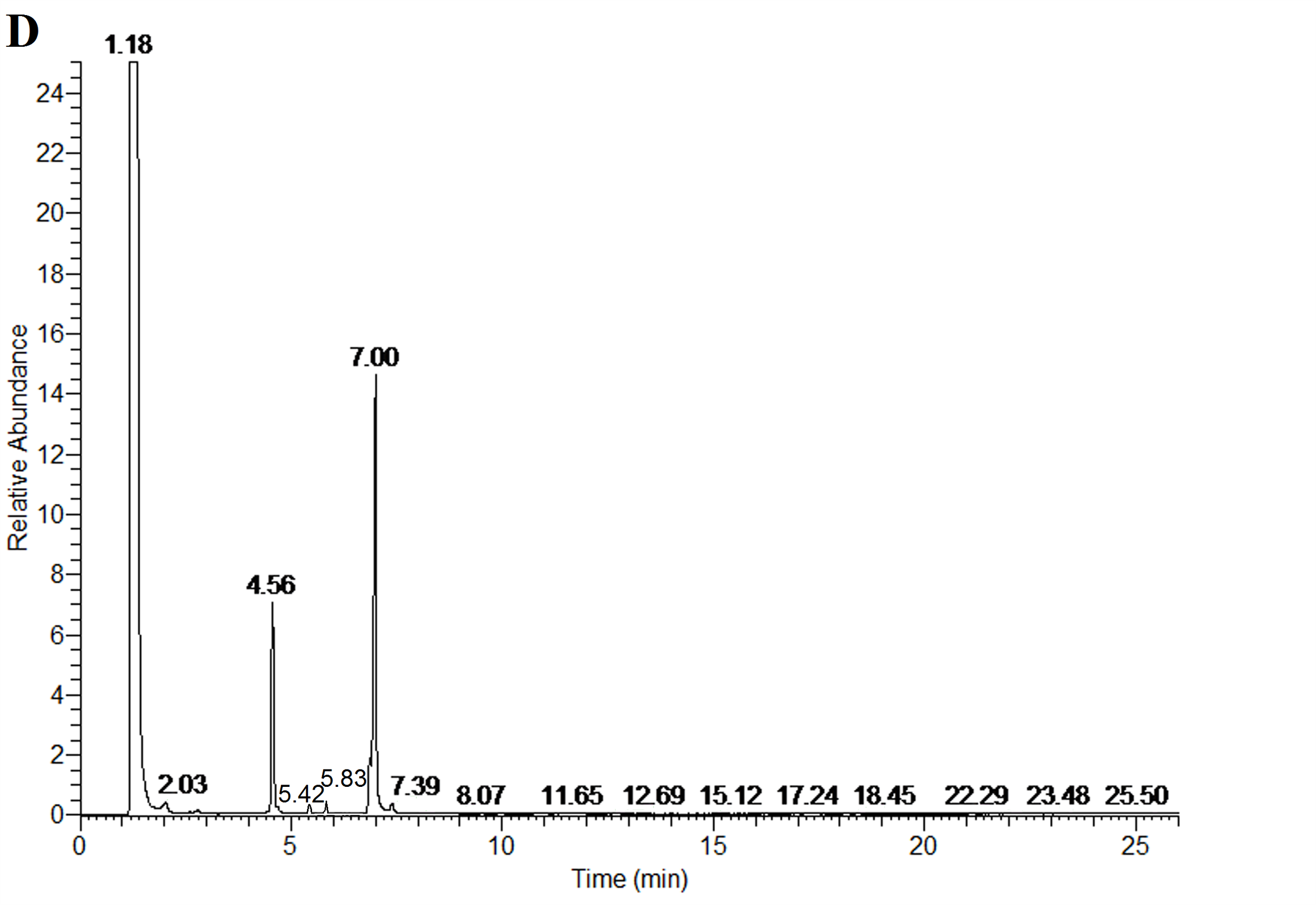


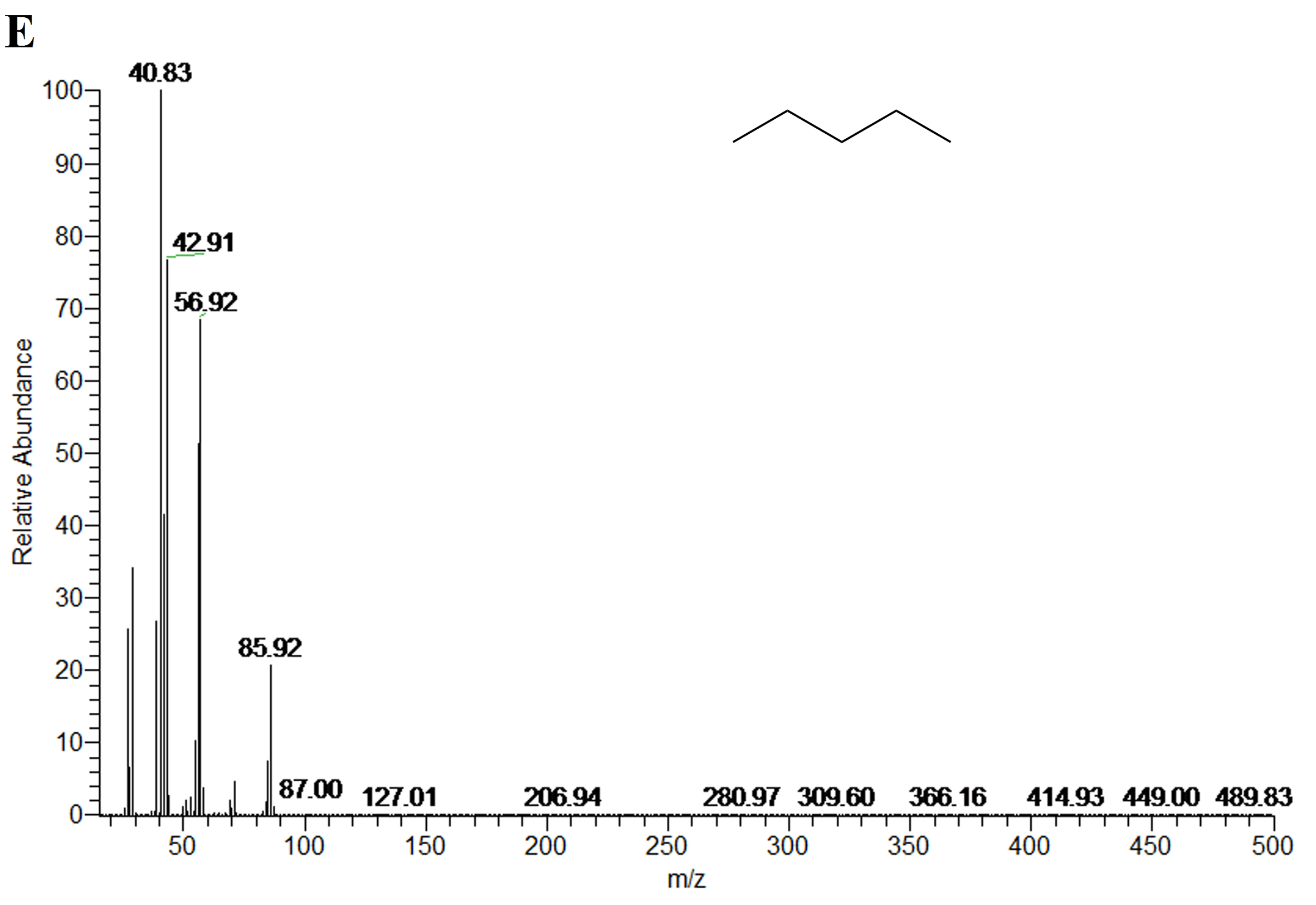


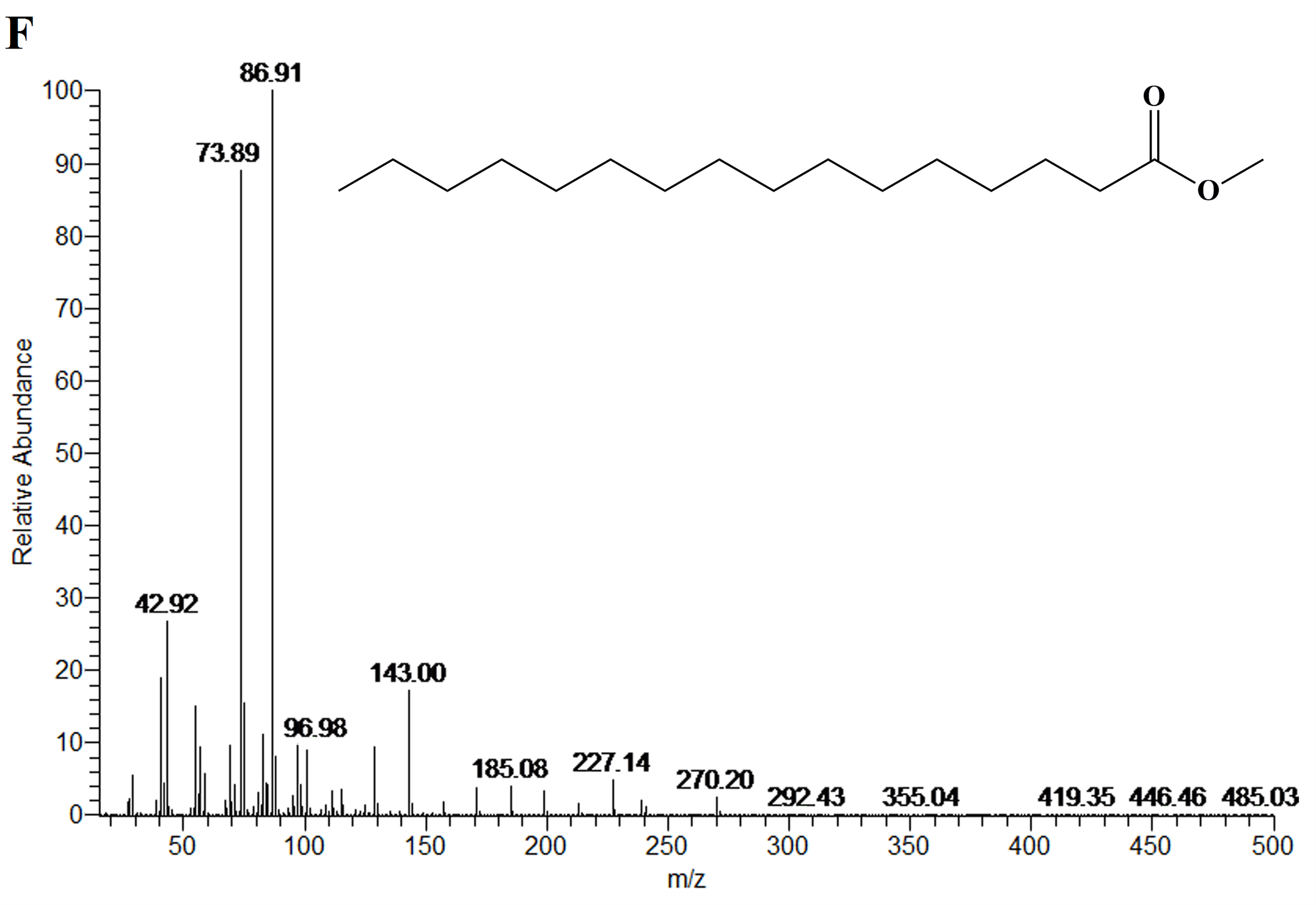


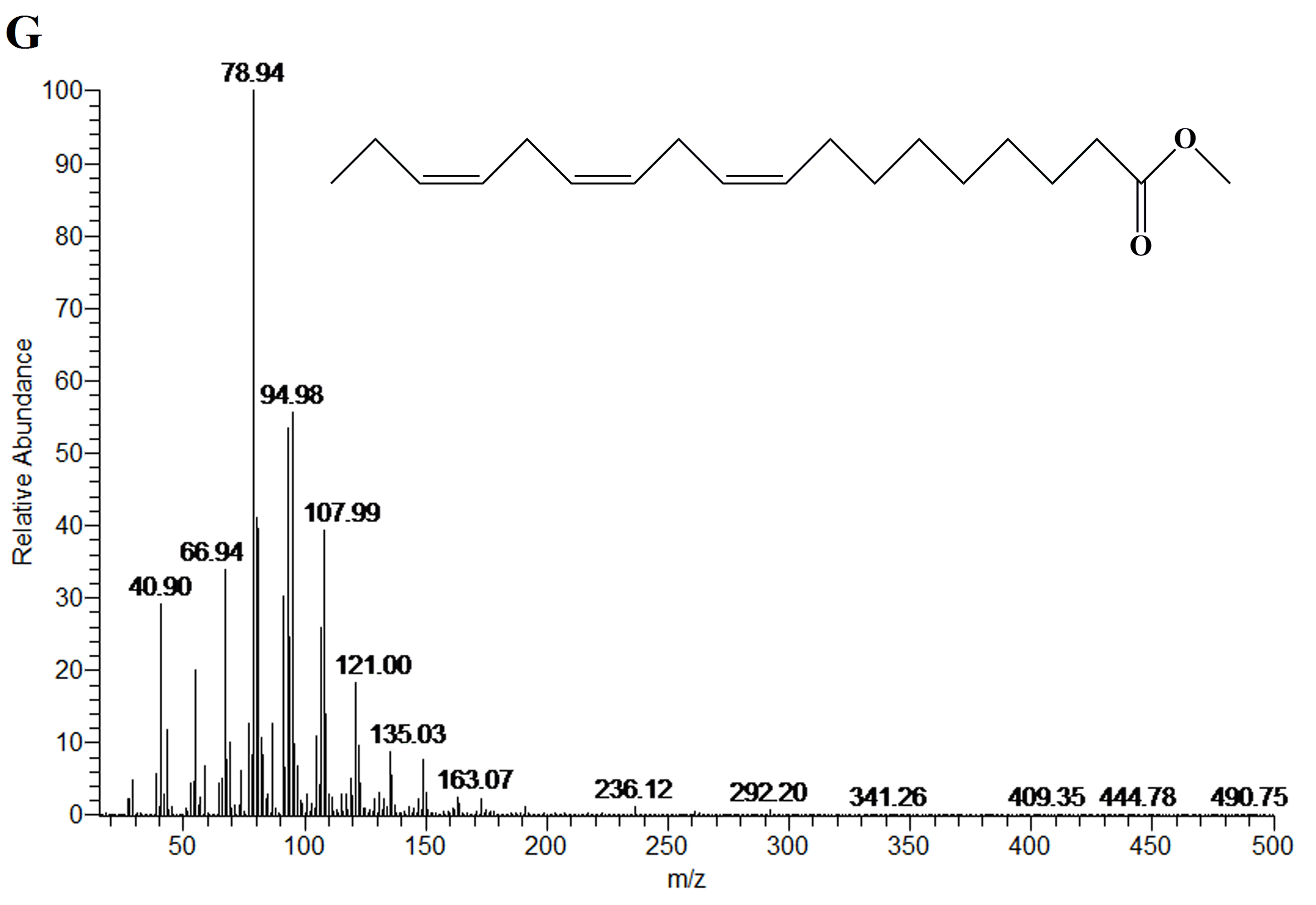


Fig. S1 The GC-MS analysis of the FAMEs of PPP and APA-style SLs. Typical GC-MS chromatogram changes of the FAMEs from PPP and APA prior to and after the reaction (A) and (D), and a representative mass spectrometry result of all species: (B) *n*-hexane (C) methyl palmitate, (E) *n*-hexane, (F) methyl palmitate, (G) methyl linolenate.
